# Supplementary material for: MicroRNA Expression Patterns Reveal a Role of the TGF-β Family Signaling in AML Chemo-Resistance
Source: Cancers (Basel). 2023 Oct 21;15(20):5086. doi: 10.3390/cancers15205086 (PMC10605523; doi:10.3390/cancers15205086)
Supplement: Supplementary file 1 [file cancers-15-05086-s001.zip › Supplementary Material.pdf]

Supplementary Material

Supplementary Table S1: Patient characteristics

Patient Nr.	Sex	Age at Diagnosis	Karyotype	Classification by ELN2016	Days until relapse	Mutation profile
**1**	**w**	**59**	46 XX	favorable	208	NPM1mut Typ A, CEBPAwt, FLT3-ITD low ratio, FLT3 TKDwt, IDH1 R132C, IDH2wt, DNMT3A R882wt
**2**	**w**	**57**	46 XX	favorable	266	biall CEBPAmut, NPM1wt, FLT3-ITD low ratio, FLT3-TKD, IDH1 und 2 wt
**3**	**m**	**38**	46,XY,del(9)(q21q32)[2]&46, XY[29]	favorable	113	biall CEBPAmut, NPM1wt, FLT3-ITD wt, FLT3-TKD wt
**4**	**w**	**35**	46,XX	intermediate	175	CEBPAwt, NPM1wt, FLT3-ITD wt, FLT3-TKD wt, IDH1 und 2 wt, DNMT3A wt

Supplementary Table S2: primer sequences for qPCR targets

Target (human)	Forward/Reverse	Sequence (5´-3´)
TGFBR1	forward	CGTCAGGTTCTGGCTCAGG
	reverse	ACAGCAACTTCTTCTCCCCG
TGFBR2	forward	TGGCTAACAGTGGGCAGGT
	reverse	GCACCAGAGCCATGGAGTAG
SMAD2	forward	AGTATGGACACAGGCTCTCCAG
	reverse	ATCGAACACCAAAATGCAGGT
SMAD3	forward	GGAGAAACCAGTGACCACCA
	reverse	GTAACTGGCTGCAGGTCCAA
SMAD4	forward	GTATCACCTGGAATTGATCTCTCA
	reverse	GATGGCTGTCCCTCAAAGTC
ACVR2A	forward	GATACCCATGGACAGGTTGGT
	reverse	TACAGCGAGAAGCCAGTTCC
ACVR2B	forward	AAGCCGTCTATTGCCCACAG
	reverse	GTCTCGTGCCTACCTGTCC
cMYC	forward	GGATTCTCTGCTCTCCTCGAC
	reverse	CTTCTTGTTCCTCCTCAGAGTC
CDKN2B	forward	GGATCCCAACGGAGTCAACC
	reverse	CACCAGCGTGTCCAGGAAG

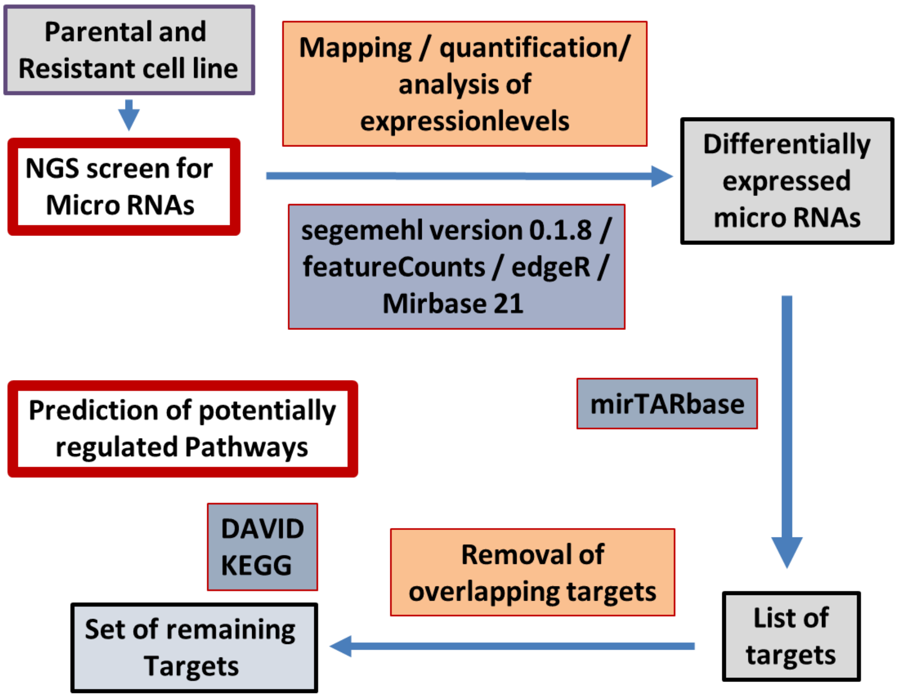

Supplementary Figure S1: Workflow and overview over the bioinformatic tools, programs and databases used for microRNA expression profiles and pathway analysis

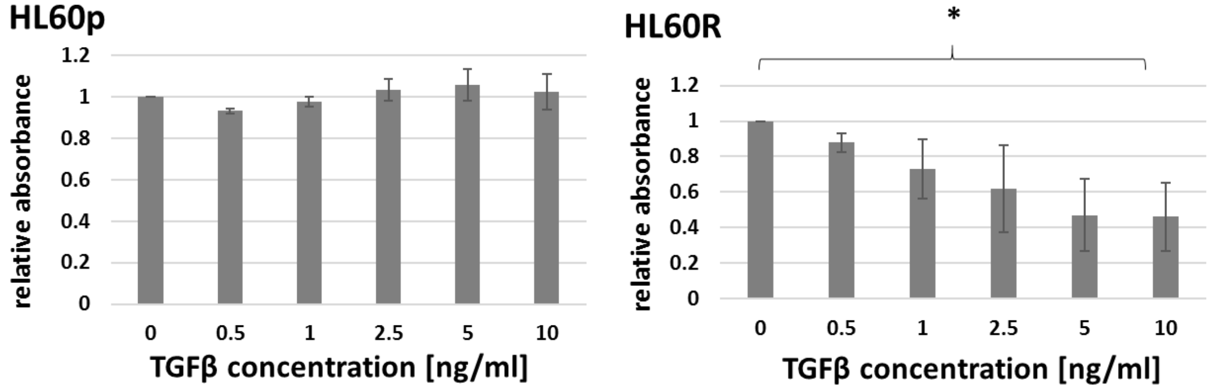

Supplementary Figure S2: Proliferation analysis; TGFβ induced growth inhibition observed by cell counts was confirmed by MTS assays. Relative absorbance of chemosensitive (left) and resistant (right) HL60 cells assessed by MTS assay at 490 nm after 3 days of treatment with different concentrations of TGFβ. Data is means ± S.E.M. from three independent replicates.

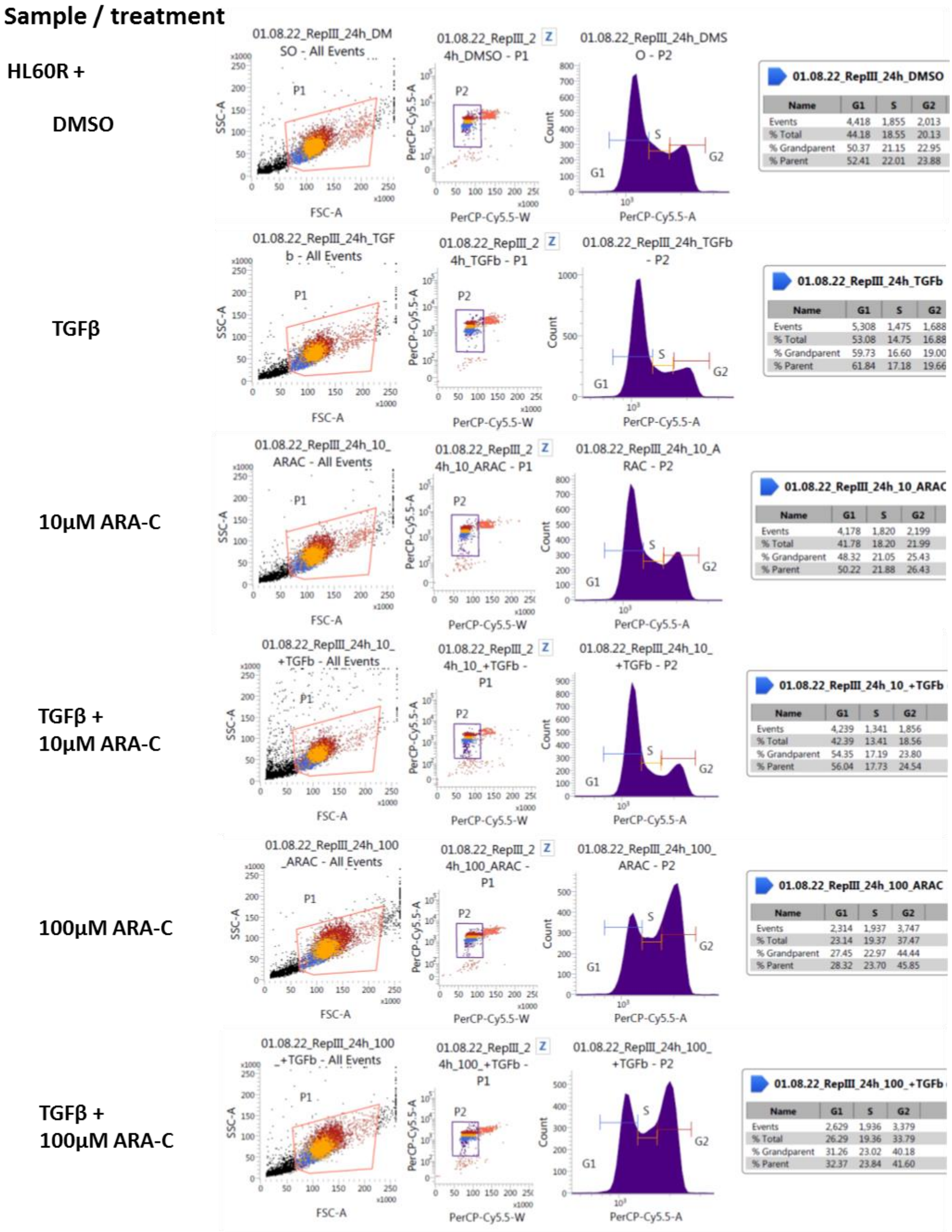

Supplementary Figure S3: Cell cycle affects of TGF-βincombinationwithdifferentconcentrationsofARA-C. Figure shows representative FACS plots of cell cycle analysis after 24 hours of treatment with different combinations of TGFβ and varying concentrations of ARA-C.

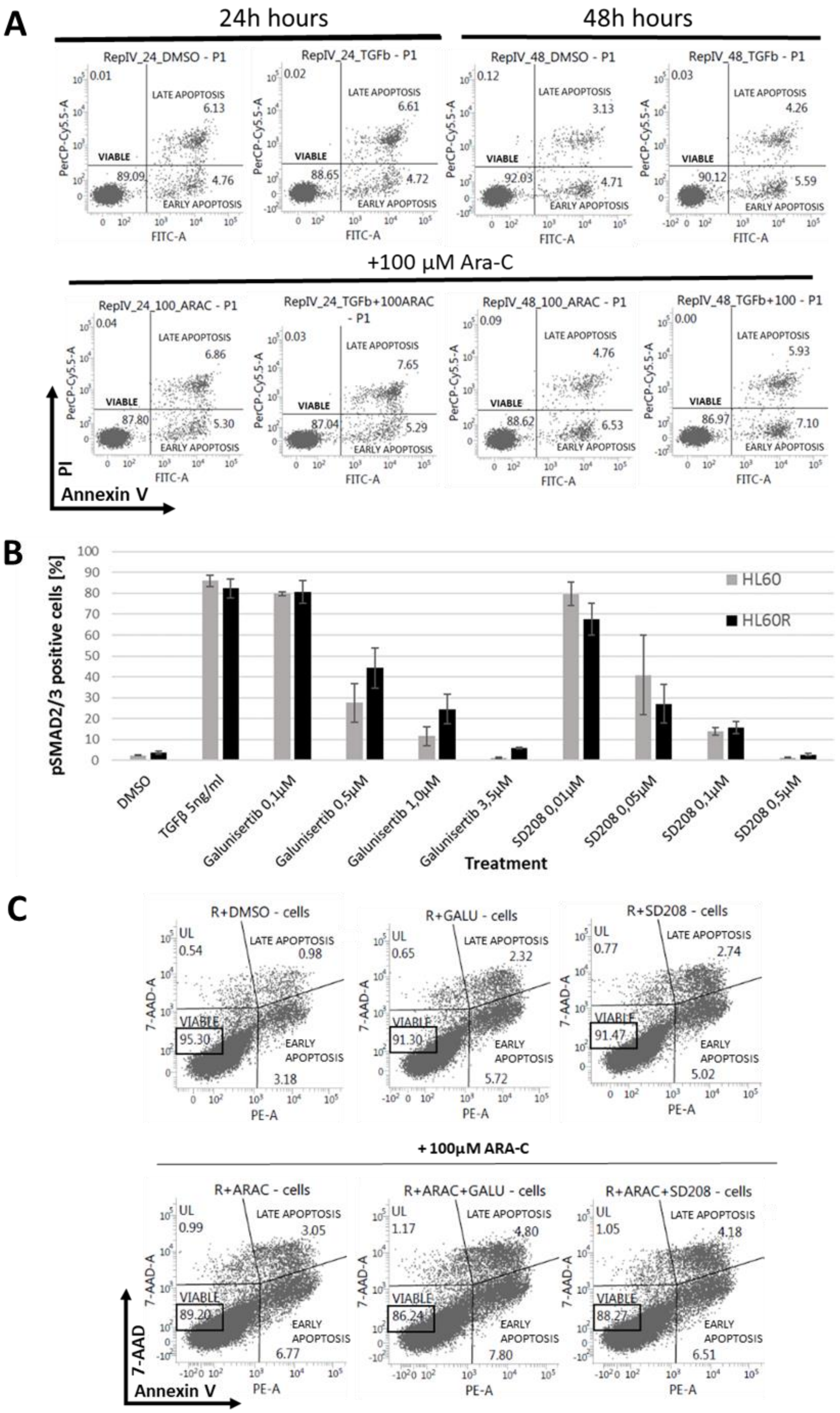
A
24h hours
48h hours
RepIV_24_DMSO - P1
RepIV_24_TGFb - P1
RepIV_48_DMSO - P1
RepIV_48_TGFb - P1
+100 µM Ara-C
RepIV_24_100_ARAC - P1
RepIV_24_TGFb+100ARAC - P1
RepIV_48_100_ARAC - P1
RepIV_48_TGFb+100 - P1
VIABLE
LATE APOPTOSIS
EARLY APOPTOSIS
PerCP-Cy5.5-A
FITC-A
0.01 6.13 89.09 4.76
0.02 6.61 88.65 4.72
0.12 3.13 92.03 4.71
0.03 4.26 90.12 5.59
0.04 6.86 87.80 5.30
0.03 7.65 87.04 5.29
0.09 4.76 88.62 6.53
0.00 5.93 86.97 7.10
PI
Annexin V
B
pSMAD2/3 positive cells [%]
HL60
HL60R
DMSO
TGFβ 5ng/ml
Galunisertib 0,1µM
Galunisertib 0,5µM
Galunisertib 1,0µM
Galunisertib 3,5µM
SD208 0,01µM
SD208 0,05µM
SD208 0,1µM
SD208 0,5µM
Treatment
C
R+DMSO - cells
R+GALU - cells
R+SD208 - cells
UL
LATE APOPTOSIS
VIABLE
EARLY APOPTOSIS
7-AAD-A
PE-A
0.54 0.98 95.30 3.18
0.65 2.32 91.30 5.72
0.77 2.74 91.47 5.02
+100µM ARA-C
R+ARAC - cells
R+ARAC+GALU - cells
R+ARAC+SD208 - cells
0.99 3.05 89.20 6.77
1.17 4.80 86.24 7.80
1.05 4.18 88.27 6.51
7-AAD
Annexin V

Supplementary Figure S4: Supplementary figure 4: A) Representative plots of flow cytometric apoptosis analysis of HL60R cells treated with DMSO (control), 100 µM ARA-C, 5 ng/ml TGF-β or combination (TGF-β + ARA-C) after 24 and 48 hours. B) Induction and pharmacological abrogation of TGFβ signaling; A) Treatment of HL60/R cells with TGF-β and TGF-β signaling inhibitors SD208 and Galunisertib; induction and abrogation of SMAD signaling was assessed by phos-flow cytometry. C) Representative Flow-cytometric apoptosis analysis of HL60R cells after over-night treatment with Galunisertib (GALU) or SD208 with and without combination with ARA-C.
