# Supplementary figures and images for: MicroRNA Expression Patterns Reveal a Role of the TGF-β Family Signaling in AML Chemo-Resistance
Source: Cancers (Basel). 2023 Oct 21;15(20):5086. doi: 10.3390/cancers15205086 (PMC10605523; doi:10.3390/cancers15205086)

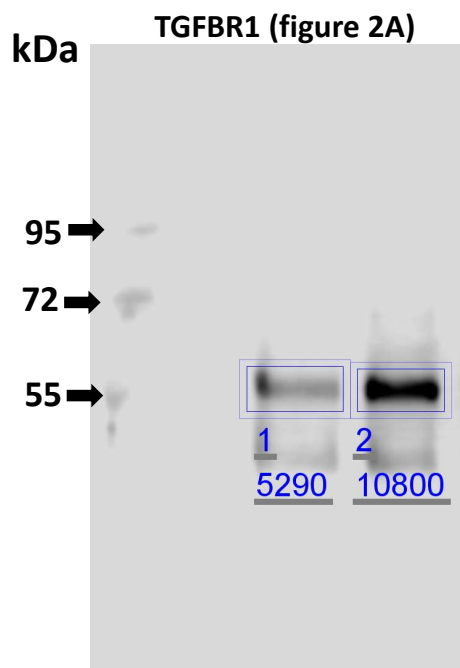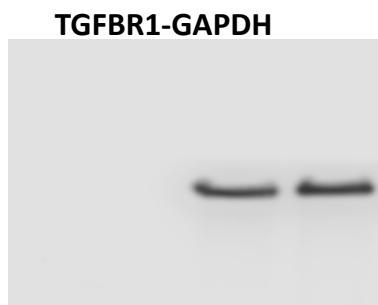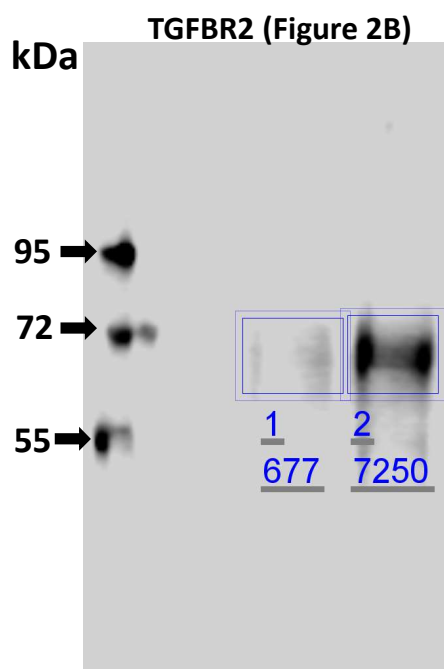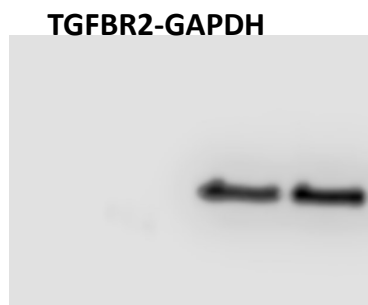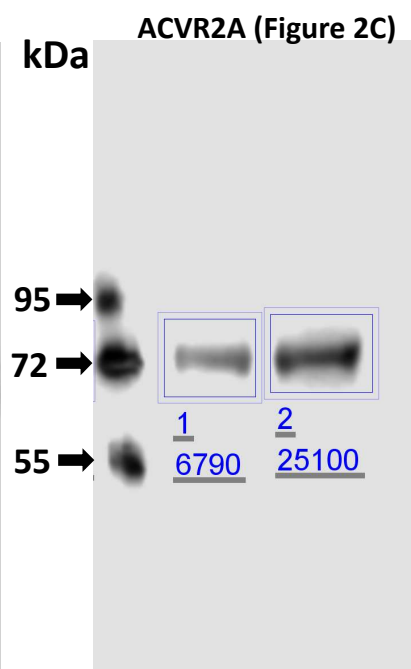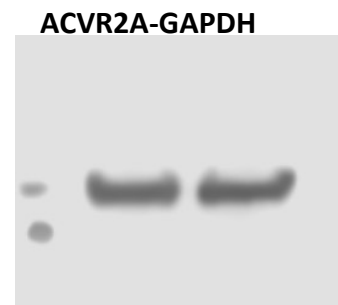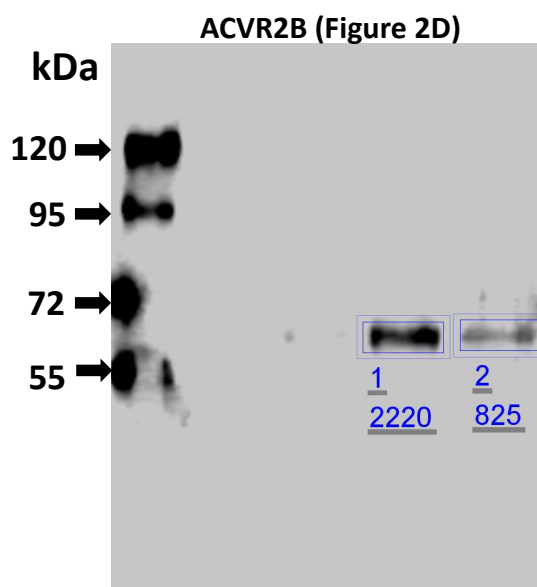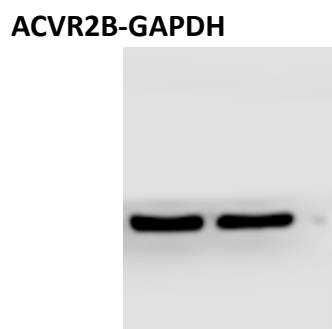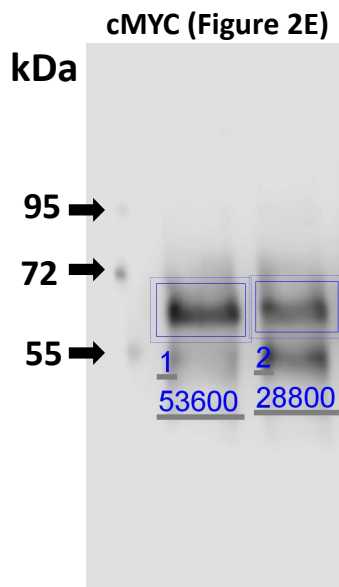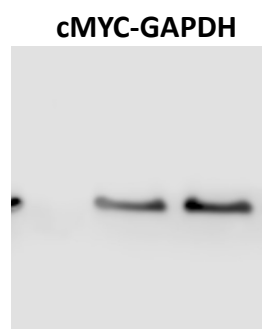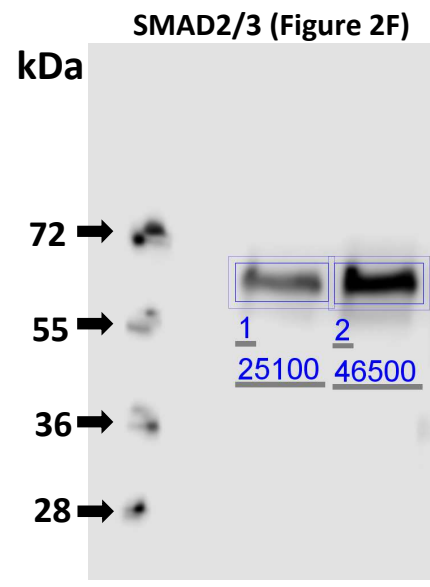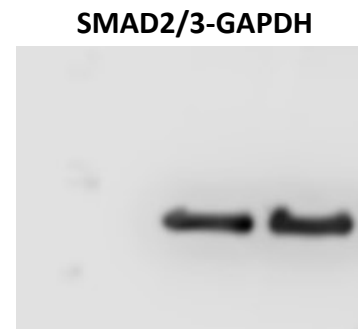

Supplement: Supplementary file 1 [file cancers-15-05086-s001.zip › File S1. WB images.pdf]
